# Aerobic and oxygen-limited naphthalene-amended enrichments induced the dominance of *Pseudomonas* spp. from a groundwater bacterial biofilm

**DOI:** 10.1007/s00253-020-10668-y

**Published:** 2020-05-16

**Authors:** Tibor Benedek, Flóra Szentgyörgyi, István Szabó, Milán Farkas, Robert Duran, Balázs Kriszt, András Táncsics

**Affiliations:** 1 Regional University Centre of Excellence in Environmental Industry, Szent István University, Páter K. u. 1, Gödöllő, H-2100 Hungary; 2 Department of Environmental Protection and Safety, Szent István University, Páter K. u. 1, Gödöllő, H-2100 Hungary; 3 IPREM UMR CNRS 5254, Equipe Environnement et Microbiologie, MELODY Group, Université de Pau et des Pays de l’Adour, Pau, France

**Keywords:** Biofilm, PAH, Naphthalene, Bioremediation, Oxygen-limited, Biobarrier

## Abstract

In this study, we aimed at determining the impact of naphthalene and different oxygen levels on a biofilm bacterial community originated from a petroleum hydrocarbon–contaminated groundwater. By using cultivation-dependent and cultivation-independent approaches, the enrichment, identification, and isolation of aerobic and oxygen-limited naphthalene degraders was possible. Results indicated that, regardless of the oxygenation conditions, *Pseudomonas* spp. became the most dominant in the naphthalene-amended selective enrichment cultures. Under low-oxygen conditions, *P. veronii/P. extremaustralis* lineage affiliating bacteria, and under full aerobic conditions *P. laurentiana*–related isolates were most probably capable of naphthalene biodegradation. A molecular biological tool has been developed for the detection of naphthalene 1,2-dioxygenase-related 2Fe-2S reductase genes of Gram-negative bacteria. The newly developed COnsensus DEgenerate Hybrid Oligonucleotide Primers (CODEHOP-PCR) technique may be used in the monitoring of the natural attenuation capacity of PAH-contaminated sites. A bacterial strain collection with prolific biofilm-producing and effective naphthalene-degrading organisms was established. The obtained strain collection may be applicable in the future for the development of biofilm-based bioremediation systems for the elimination of PAHs from groundwater (e.g., biofilm-based biobarriers).

**Electronic supplementary material:**

The online version of this article (10.1007/s00253-020-10668-y) contains supplementary material, which is available to authorized users.

## Introduction

Polycyclic aromatic hydrocarbons (PAHs), originating either from natural or from anthropogenic sources, are widespread persistent and toxic pollutants of great concern. Many PAHs, consisting of two or more combined benzene rings, are known to be mutagenic and/or carcinogenic (Harvey 1991). The US Environmental Protection Agency (EPA) has listed 16 PAHs (including naphthalene) as priority pollutants (Cerniglia and Heitkamp 1989). PAHs can enter to the environment via several routes including the burning of fossil fuels, processing of gas/coal tar/wood, waste incineration, and fuel leakage (for a review, see Duran and Cravo-Laureau 2016). Apart from acenaphthene, acenaphthylene, and dibenz[a,h]anthracene, which are primarily detected in gasoline and diesel exhaust gas, the majority of the 16 priority pollutant PAHs (13) can be detected in gasoline itself (Zoccolillo et al. 2000; NCBI PubChem 2020).

Microbial degradation is the major mechanism in determining the fate of PAHs in the environment, whether in aquatic (e.g. marine, Cravo-Laureau and Duran 2014) or terrestrial ecosystems (Ghosal et al. 2016). Therefore, microbial resources have been considered for the eco-friendly remediation of PAH-contaminated sites (de Boer and Wagelmans 2016; Bordenave et al. 2004b; Ben Said et al. 2015).

Today, more and more efforts are being made globally on the improvement of sustainable PAH bioremediation strategies. Compared to the physical-chemical approaches, the biological treatment of contaminated sites is a more preferred, efficient, and cost-effective choice (Kuppusamy et al. 2016). Biodegradation of PAHs leads to the complete degradation of the pollutants with greater safety and with reduced environmental disturbance (Habe and Omori 2003). Studies dealing with PAH biodegradation should point in the direction of sustainability from both environmental and financial aspects. Therefore, based on global trends and taking into consideration sustainability, a better understanding of microbial communities responsible for efficient PAH biodegradation under different environmental circumstances—e.g., aquatic versus terrestrial ecosystem, marine versus freshwater environment, aerobic/oxygen-limited/anaerobic conditions—is a must. Related to the oxygenation conditions, our previous studies already pointed out the importance of available oxygen in petroleum hydrocarbon–contaminated environments. As it was found earlier, the presence of enough available oxygen in the contaminated environment could substantially increase the biological degradation rate of petroleum hydrocarbons (crude oil or PAHs). Oxygenation proved to be critical in the process of biodegradation. It was also observed that oxic/anoxic oscillations can be as effective as full aerobic conditions during hydrocarbon biodegradation. However, it has to be added that oxic/anoxic alternations may have an undesired impact upon bacterial community structures, influencing their ability to degrade hydrocarbons and their capacity to reduce hydrocarbon toxicity (Cravo-Laureau et al. 2011; Vitte et al. 2011; Duran et al. 2015; Militon et al. 2015; Terrisse et al. 2017). It was also found previously that, in subsurface hydrocarbon-contaminated groundwater, oxygen availability is one of the main driving factors in the organization of microbial communities (Benedek et al. 2016, 2018).

The isolation of efficient PAH-degrading bacteria, applicable in the development of innovative, relatively cheap PAH remediation technologies, like biobarriers, small bioreactor platform technology SBPs, enzyme-mediated bioremediation, and mixed cell culture systems—algal/bacterial consortia, etc.—has become also important (Carreghini et al. 2013; Kuppusamy et al. 2016; Menashe and Kurzbaum 2014). As we previously demonstrated, biofilms/microbial mats, developed in hydrocarbon-contaminated environments, represent a microbial resource from which microorganisms with the desired bioremediation potential can be isolated (Bordenave et al. 2004a; Benedek et al. 2016, 2018). In our previous studies, the targeted biofilms (Bugyi site, freshwater environment, Hungary) and microbial mats (Camargue site, marine environment, France) proved to be the habitat of complex networks of chemoorganotrophic and chemolithotrophic bacteria where microorganisms with opposite traits (e.g., aerobic/anaerobic, iron reducers/iron oxidizers) co-existed and interacted on complementary processes (Benedek et al. 2016). The collected samples proved to be the reservoir of hydrocarbonoclastic bacteria capable of aerobic and oxygen-limited (hypoxic) degradation of simple aromatic hydrocarbons (BTEX), as well as low-molecular-weight (LMW < 4 rings) PAHs. Moreover, the investigated biofilm samples also proved to be the source of catabolic genes involved in aerobic (I.2.A and I.2.B catechol 2,3-dioxygenases), microaerobic (I.2.C catechol 2,3-dioxygenases) and anaerobic (*bssA*) simple aromatic hydrocarbon degradation, as well as in LMW-PAH biodegradation (Bordenave et al. 2004a; Benedek et al. 2016, 2018).

In this study, we aimed at determining the impact of naphthalene (LMW-PAH) on a biofilm bacterial community, which developed initially in hydrocarbon-contaminated groundwater at the Bugyi site (Hungary), where the concentration of oxygen is generally low. Based on our previous studies (Benedek et al. 2016, 2018), we believe that the investigated biofilm, with high phylogenetic and functional diversity, is an accurate and easy-to-handle microbial community to investigate the impact of PAHs (e.g., naphthalene) on bacterial communities under different oxygen levels. It is hypothesized that distinct aerophilic conditions will shape the versatile bacterial community and induce the emergence of bacterial genera adapted to aerobic and oxygen-limited degradation of PAHs. To test the hypothesis, naphthalene will be used as a model PAH compound. We also expect to isolate PAH-degrading and biofilm-producing bacteria, which can be useful for the development of semipermeable biofilm-based biobarriers. Overall, our results provide valuable information regarding PAH biodegradation in hypoxic subsurface freshwater ecosystems.

## Materials and Methods

### Site description, pollution history, and biofilm sampling

The bacterial biofilm sample was collected in January 2018 in the Central Region of Hungary. The sample was taken from the stainless steel surface of a submersible pump, which belonged to a Pump and Treat system (P&T system) treating gasoline-contaminated groundwater. In 2007, due to an accident, ~ 27 m^3^ of gasoline leaked into the shallow groundwater leading to aliphatic and aromatic hydrocarbons contamination of the sampling site. Within the contamination plume, the installation of a rapid in situ decontamination P&T system was crucial for ecological and public health reasons. On the date of system startup, the main groundwater pollutants were BTEX—benzene (7 mg l^−1^), toluene (28.5 mg l^−1^), ethyl-benzene (1 mg l^−1^), xylenes (10.4 mg l^−1^)—and total aliphatic petroleum hydrocarbons (TAPH C_5_–C_40_, 10 mg l^−1^). For more details regarding site characterization and P&T system operation, please see Benedek et al. (2016, 2018).

Although the concentration of PAHs has not been determined separately, based on literature data, their presence in gasoline-contaminated sites is highly expected. PAHs are a class of chemicals that occur naturally in gasoline (Candeli et al. 1975; Zoccolillo et al. 2000). Marr et al. (1999) by measuring the PAH content of five different gasoline samples (regular, premium, and midgrade quality) found that on average, naphthalene contributes 97 ± 1% of the total concentration of the PAHs measured. PAH concentrations in the fuels ranged from undetectable (< 0.1 mg l^−1^) for several of the higher molecular weight PAHs to 2600 mg l^−1^ for naphthalene. Gasoline’s naphthalene content varies by brand and grade, and “premium” gasoline tends to have higher concentrations than “regular” gasoline (Jia and Batterman 2011). Naphthalene content of gasoline has been expressed as 1.04 mg g^−1^ (Schauer et al. 2002), 69 to 2600 mg l^−1^ (Marr et al. 1999), and 0.15 to 0.18% (w/w) (Harley et al. 2000).

The temperature of slightly alkaline (pH 7.3–7.5) groundwater is between 10 and 14 °C. The oxygen concentration of the groundwater is in the hypoxic range (≤ 2 mg l^−1^). The treatment capacity of the system is 30 m^3^ of contaminated groundwater per day. Owing to the P&T system, the groundwater has been gradually decontaminated. By the time of sampling, in the case of the biofilm sampling well BUT18, the concentration of the abovementioned hydrocarbons (BTEX and TAPH) had decreased significantly below the threshold values defined by the Hungarian Government, Regulation No. 6/2009. (IV.14) KvVM-EÜM-FVM.

In the aforementioned P&T system, on the surface of submersible pumps located in the extraction wells and inside the pipelines, frequent bacterial biofilm formations were observed. For our studies, the biofilm sample was collected from the extraction well BUT18 and placed into sterile 50-ml Falcon conical centrifuge tubes. Samples were stored in dry ice and were processed immediately upon arrival at the laboratory.

### Enrichment of naphthalene-degrading biofilm bacteria

Naphthalene-degrading enrichment cultures were similarly initiated as described previously in the case of BTEX-degrading biofilm enrichments (Benedek et al. 2018).

In 100-ml crimp-sealed serum bottles, in mineral salt solution (49 ml) supplemented with vitamins (Fahy et al. 2006), full aerobic (~ 8 mg l^−1^ O_2_) and oxygen-limited (≤ 0.5 mg l^−1^ O_2_) enrichment microcosms were set up in duplicates. One milliliter of biofilm suspension (1 g of biofilm suspended in 9 ml mineral salt solution) was added to each microcosm as an inoculant. Naphthalene crystals were added to the microcosms in a final concentration of 100 mg l^−1^ as the only source of carbon and energy (Acros Organics, Belgium). After 1 week of incubation (28 °C at 150 rpm), the naphthalene crystals, which were initially clearly visible to the naked eye, totally disappeared from the aerobic enrichments. Consequently, 1 ml of the aerobic enrichment was transferred to freshly prepared enrichment medium amended again with naphthalene crystals. Six consecutive transfers were conducted. In the case of oxygen-limited enrichments, the transfers were done every 2 weeks, three transfers in total. For the oxygen-limited bacterial community, 2 weeks were needed for the complete degradation of the naphthalene crystals. Before initiating the oxygen-limited enrichments, microcosms were sparged aseptically with N_2_/CO_2_ (80:20, *v/v*) for 10 min. The desired oxygen concentration (0.5 mg l^−1^) was set by sterile air injection (0.2-μm-pore-size-filtered) through the butyl-rubber septa. Oxygen concentration in the liquid phase was measured non-invasively by using a Fibox 3 trace v3 fiber optic oxygen meter with PSt3 sensor spots (PreSens, Regensburg, Germany). In the case of oxygen depletion, supplementation was performed.

Indirect evidence, namely naphthalene biodegradation screening tests of isolates prior to GC-MS measurements (data not shown), suggest that during the enrichment, naphthalene crystals’ disappearance from the bottles was predominantly due to biodegradation and not to volatilization.

### Isolation of naphthalene-degrading bacteria

The isolation of naphthalene-degrading bacteria was performed in the case of both aerobic and oxygen-limited enrichments. Bacterial isolations occurred during the first transfer (after 1 or 2 weeks of incubation) and at the end of the enrichment period (after 6 weeks of incubation).

Prior to isolation, from the respective microcosms, 1 ml was used for the preparation of tenfold serial dilutions in physiological salt solution (0.9 % m/v; up to 10^−6^ dilution). One hundred microliters of serially diluted aerobic and oxygen-limited samples was spread on the surface of (i) R2A agar plates, (ii) R2A agar plates amended with naphthalene, or (iii) Bushnell-Haas-Broth plates solidified with gellan gum and supplemented with naphthalene. The composition per liter of R2A agar was the following: proteose peptone 0.5 g, casamino acids 0.5 g, yeast extract 0.5 g, dextrose 0.5 g, soluble starch 0.5 g, dipotassium phosphate 0.3 g, MgSO_4_·7H_2_O 0.05 g, sodium pyruvate 0.3 g, agar 15 g, pH 7 ± 0.2. Bushnell-Haas-Broth mineral salt medium was contained in 1000 ml of H_2_O 0.002 g of CaCl_2_·2H_2_O, 1 g of MgSO_4_·7H_2_O, 1 g of NH_4_NO_3_, 1 g of KH_2_PO_4_, 1 g of K_2_HPO_4_, 0.005 g of FeCl_3_·6H_2_O, and 8 g of gellan gum, and the pH was set to 7 ± 0.2. Naphthalene had been spread on the surface of solidified media in the form of naphthalene-methanol solution (100 μl, 10 g l^−1^). After methanol evaporation, white crystals of naphthalene appeared and then inoculation of the bacterial suspension took place (100 μl of each member of the tenfold dilutions). After inoculation, Petri dishes were thoroughly sealed with parafilm to reduce evaporation of naphthalene. Plates were incubated at 15 °C for 2 weeks (incubation temperature common to in situ conditions of the groundwater). Developed colonies, showing different morphologies, were purified by streak plating and maintained on R2A agar slants at 4 °C, as well as at − 80 °C in a glycerol solution (15% v/v). All chemicals, if not stated otherwise, were purchased from Sigma-Aldrich, Germany.

### Extraction of nucleic acids

Total community DNA from the biofilm sample (0.5 g) was extracted by using the DNeasy® PowerSoil Kit (Qiagen, Hilden, Germany). Community DNA of the enrichment cultures and genomic DNA of the isolates were extracted by using the DNeasy® UltraClean® Microbial Kit (Qiagen, Hilden, Germany). In the case of microcosms, 49 ml of enrichment cultures was centrifuged (2360×*g*, 15 min, Rotanta 460 R centrifuge—Hettich, Germany) and the DNA was extracted from the pellet. Genomic DNA isolation was performed from overnight cultures of bacteria. The manufacturer’s instructions were followed in all cases.

### Gene amplifications

The species-level identification of cultivable biofilm bacteria took place on the basis of *16S rRNA* gene–based sequence analysis. PCR amplification of the gene was performed as described earlier. Universal bacterial primers 27F (5′-AGAGTTTGATC(A/C)TGGCTCAG-3′) and 1492R (5′-TACGG(C/T)TACCTTGTTACGAC TT-3′) were used (Benedek et al. 2016).

For Illumina paired-end *16S rRNA* amplicon sequencing, the variable V3 and V4 regions of the gene were amplified as described in our previous study (Benedek et al. 2018). The forward and reverse primers with Illumina adapter overhanging nucleotide sequences (written in bold) were the following: forward 5′-**TCGTCGGCAGCGTCAGATGTG TATAAGAGACAG**CCTACGGGNGGCWGCAG-3′ and reverse 5′-**GTCTCGTGGGCT CGGAGATGTGTATAAGAGACAG**GACTACHVGGGTATCTAATCC-3′ (Klindworth et al. 2013). PCR conditions and reagent concentrations were the same as described earlier (Benedek et al. 2018).

### CODEHOP primer design for the detection of NDO

A new set of COnsensus DEgenerate Hybrid Oligonucleotide Primers has been designed to assess the PAH biodegradation ability of the initial biofilm community, and the enriched cultures, as well as of the isolated strains (CODEHOP, Rose et al. 2003, Staheli et al. 2011). During the primer design, we focused on the reductase component of the naphthalene 1,2-dioxygenase gene found in Gram-negative bacteria (NDO; EC 1.14.12.12). NDO is a member of the ring-hydroxylating dioxygenase (RHD) family of bacterial enzymes that play a pivotal role in the degradation of aromatics, such as PAHs (naphthalene, fluorene, anthracene, and benzo[a]pyrene). The enzyme is comprised of a multicomponent system, containing a reductase that is an iron-sulfur (2Fe-2S) flavoprotein (FAD; EC 1.18.1.3, ferredoxin-NAD^+^ reductase), an iron-sulfur oxygenase, and ferredoxin (Ensley and Gibson 1983; Jouanneau et al. 2006).

Primer design was done on the basis of conserved blocks of amino acids within aligned 2Fe-2S reductase protein sequences. Amino acid sequences were retrieved from the GenBank and aligned with ClustalW (MEGA version 7.0). For primer design, the aligned multiple protein sequences were subjected to j-CODEHOP design software, an integrated tool into Base-by-Base (Tu et al. 2018). The length of the non-degenerate 5′ clamp region was set to 18 base pairs (bp). The length of degenerate 3′ region was set to 4 amino acids (aa). Max degeneracy was set to 64, while min AA conservation to 80%.

Amplifications with the newly obtained CODEHOP primers were performed in 50 μl reaction mixture. The mixture contained 5 μl 10x DreamTaq^TM^ buffer (ThermoFisher Scientific, Lithuania) with MgCl_2_ (2 mM), 0.2 mM of each dNTP, 0.1 μM of each primer, 1 U DreamTaq^TM^ DNA Polymerase (ThermoFisher Scientific, Lithuania), 1 μl (~ 40 ng) extracted DNA, and nuclease-free water up to the final reaction volume. The following amplification conditions were applied: 95 °C for 3 min, then 32 cycles of 94 °C for 30 s, annealing temperature 66 °C for 30 s and 72 °C for 1 min, and then a final extension at 72 °C for 10 min. Amplifications were conducted on (i) isolate-derived genomic and (ii) community-derived DNA samples (initial biofilm and enrichment samples). PCR amplifications were expected to yield PCR products of ~ 750 bps. To check the specificity of the CODEHOP-primer pair for PCR amplification of the target sequence, positive and negative control genomic DNA samples were used. According to the whole genome sequences, *Z. oleivorans* strain BUC-1^T^ (SDKK00000000), *R. pyridinivorans* AK37 (NZ_AHBW00000000), and *C. basilensis* OR16 (NZ_AHJE00000000) do not have NDO-related 2Fe-2S reductase genes in their genomes. Therefore, genomic DNA originating from those isolates was used as negative control. Since *Malikia spinosa* strain AB6 contains NDO reductase in its genome (VYSV00000000), its genomic DNA was used as a positive control during the PCR amplifications. As a further act of validation, all CODEHOP PCR products (originating either from the community or from isolate DNA) were sequenced and homology BLAST searches were made in the NCBI GenBank database.

All amplifications were carried out in a ProFlex PCR System (Applied Biosystems by Life Technologies, USA). Amplicons were analyzed under UV light after electrophoresis in 1% (w/v) agarose gel stained with EtBr. All amplicons, prior to subsequent analyses, were purified by NucleoSpin® Gel and PCR Clean-up set (Macherey-Nagel, Düren, Germany).

### Cloning of NDO reductase component genes

The diversity of NDO-related 2Fe-2S reductase genes, from aerobic and oxygen-limited enrichment samples of the sixth week, was assessed by molecular cloning as described by Táncsics et al. (2012). The ~ 750-bp-long PCR products were cloned into pCR 2.1 vector (Invitrogen, Carlsbad, USA) following the manufacturer’s instructions. Standard blue-white selection method was applied for the selection of transformants. Plasmid DNA was extracted with heat shock (98 °C, 5 min); inserts were reamplified using M13 primer set (forward 5′-GTA AAA CGA CGG CCA G-3′, reverse 5′-CAG GAA ACA GCT ATG ACC-3e).

### DNA sequencing

Illumina 16S rDNA amplicon sequencing was performed to compare the bacterial community composition of the initial biofilm to the selected aerobic (NAF_A_A.6) and oxygen-limited enrichments (NAF_H_A.6) of the final week. Illumina 16S rDNA (V3-V4 region) sequencing was performed as described previously (Benedek et al. 2018). Primary data analysis (base-calling) was carried out with Bbcl2fastq^ software (v2.17.1.14, Illumina). Reads were quality and length trimmed in CLC Genomics Workbench Tool 9.5.1 using an error probability of 0.05 (Q13) and a minimum length of 50 nucleotides as threshold. Trimmed sequences were processed using mothur v1.41.1 (Schloss et al. 2009) as recommended by the MiSeq SOP page (http://www.mothur.org/wiki/MiSeq_SOP) (Kozich et al. 2013). Sequences were assorted based on the alignment using SILVA 132 SSURef NR99 database (Quast et al. 2013). Chimera detection was performed with mothur’s uchime command (Edgar et al. 2011), and ‘split.abund’ command was also used to remove singleton reads according to Kunin et al. (2010). The standard 97% similarity threshold was used to determinate operational taxonomic units (OTUs) as suggested by Tindall et al. (2010) for prokaryotic species delineation. Raw sequence reads were deposited in NCBI SRA under BioProject ID PRJNA562625. Abundant OTUs were also identified by applying EzBioCloud 16S rDNA database (Yoon et al. 2017).

The nucleotide sequence determination of *16S rRNA* and NDO-related 2Fe-2S reductase genes was performed by Sanger sequencing using BigDye Terminator v3.1 Cycle Sequencing Kit (Life Technologies, USA). Sequences were analyzed with ABI 3130 Genetic Analyzer (Life Technologies, USA), edited and assembled using MEGA version 7.0 (Kumar et al. 2016). Homology BLAST searches (Altschul et al. 1997) were made in the GenBank database (http://www.ncbi.nlm.nih.gov/BLAST/). On the basis of *16S rRNA* genes, EzTaxon-e server carried out the determination of the closest type strains of isolates (http://eztaxon-e.ezbiocloud.net/; Kim et al. 2012). For DNA sequencing, both forward and reverse sequencing primers were used. In the case of NDO-related 2Fe-2S reductase genes for the sequencing reaction, a new set of CODEHOP primers was used without the degenerate core.

*16S rRNA* gene sequence data obtained in this study was deposited in the GenBank under the accession numbers MN197554-MN197593. Putatively NDO-related 2Fe-2S reductase component gene sequences derived from uncultured bacteria were deposited in the GenBank under the accession numbers MN370456-MN370503 (aerobic enrichment) and MN370504-MN370547 (oxygen-limited enrichment). NDO-related reductase component gene sequences, which were derived from the biofilm-related bacterial isolates, were deposited under the accession numbers (MN420520-MN420545).

### Temporal dynamics assessed by T-RFLP

Terminal restriction fragment length polymorphism (T-RFLP, Liu et al. 1997) was used to assess the alterations of the initial biofilm community throughout the whole enrichment period. For phylogenetic T-RFLP analysis, *16S rRNA* genes from the initial biofilm and from the enrichments DNA were PCR amplified by using VIC fluorescently labelled 27F and non-labelled 1492R primers. Reagent concentrations and PCR conditions were the same as described earlier (Benedek et al. 2014). The obtained PCR products were digested with 1 U *Alu*I (AG↓CT) restriction endonuclease (Fermentas, Lithuania) for 1.5 h at 37 °C. The generated fluorescently-labelled terminal restriction fragments (T-RFs) were purified by ethanol precipitation. Fragments were separated by capillary gel electrophoresis. T-RFs were detected using a Model 3130 Genetic Analyzer (Applied Biosystems, USA). GeneScan^TM^ 1200 LIZ^TM^ internal size standard was used (Applied Biosystems, USA). The resulted T-RFLP electropherograms were analyzed with the GeneMapper Software version 4.0 (Applied Biosystems, USA). For consensus profiles, runs of two replicates were aligned with the T-Align program and 0.5-bp confidence interval was used (Smith et al. 2005).

To determine temporal dynamics of the initial biofilm community, due to the different enrichments, the T-align originated comparison results files were evaluated with the PAST software (Hammer et al. 2001). PCA (principal component analysis) was used to visualize the results.

### Testing naphthalene degradation potential of biofilm isolates by GC-MS

Naphthalene biodegradation ability of isolates was assessed through microcosm experiments by gas chromatography coupled to mass spectrometry (GC-MS). Microcosms were set up in triplicates in hermetically closed, crimp-sealed vials containing 50 ml BBH mineral salt solution and naphthalene in a final concentration of 1 mg l^−1^. Naphthalene was introduced into the bottles in the form of methanol-naphthalene stock solution (1 g l^−1^) in a 50-μl volume. Microcosms were inoculated with 100 μl of bacterial cell suspensions made in physiological saline solution (OD_600nm_ = 1). The concentration of naphthalene at the beginning of the experiment, as well as after 20 h of incubation (28 °C, 150 rpm), was determined from the headspace using an SPME polydimethylsiloxane fiber assembly (Supelco) for sampling and a Trace 1300 gas chromatograph coupled to ISQ Single Quadrupole mass spectrometer (Thermoscientific) for analysis. During the analysis, injector and detector temperatures were maintained at 240 °C and 270 °C, respectively. The oven temperature program was set to 40 °C for 3 min and then ramped at a rate of 70 °C min^−1^ to 230 °C and finally held for 5 min. Helium was used as the carrier gas at a flow rate of 1.2 ml min^−1^. SLB TM − 5 ms Fused Silica capillary column was used for separation (30 m × 0.25 mm × 0.25 μm, Sigma-Aldrich, Supelco). The mass spectrometer (MS) operated at full scan mode.

### Testing biofilm-forming ability of isolates

Biofilm-forming ability of isolated strains was determined as described in our previous study (Benedek et al. 2018). Briefly, 250 μl bacterial cell suspensions in R2A medium were inoculated in triplicates into chimney well cell culture polystyrene microplates (greiner bio-one, Germany). After incubation at 28 °C for 24, 48, and 72 h, wells were decanted and rinsed three times with phosphate buffer (250 μl). Subsequently, biofilms that adhered to well surfaces were fixed with methanol (99%, 250 μl) and stained with crystal violet (0.5%, 250 μl). After cell resuspension with 33% glacial acetic acid, the absorbance of the solution (*A*) at 550 nm (absorbance of crystal violet) was determined by using a BioTek ELx800 Absorbance Microplate Reader (BioTek, USA). Evaluation of the results was done according to Stepanović et al. (2000):

- *non*-*adherent A*_sample_ ≤ *A*_C_
- *weakly adherent A*_C_ < *A*_sample_ ≤ 2 × *A*_C_
- *moderately adherent* 2 × *A*_C_ < *A*_sample_ ≤ 4 × *A*_C_
- *strongly adherent* 4 *A*C < *A*_sample_

*A*_C_ represents the cutoff absorbance value defined as three standard deviations above the mean absorbance of the negative control.

## Results

### Bacterial diversity as assessed by cultivation-dependent technique

From the enrichment cultures, a set of 40 bacterial strains were isolated (Table 1). Irrespective of the isolation method, the majority of the isolates belonged to the genus *Pseudomonas* (75%). The highest number of *Pseudomonas* spp.–related strains was obtained during the last isolation step (20 isolates). In the aerobic enrichment, *Pseudomonas* spp.–related strains mostly affiliated with *P. silesiensis* (1st week) and *P. umsongensis* (6th week). Mainly *P. veronii*–related strains were obtained from the oxygen-limited enrichments. *Pseudomonas* spp. were followed by *Acidovorax* (4 isolates, *Gammaproteobacteria*), *Rhodococcus* (3, *Actinobacteria*), *Achromobacter* (2, *Gammaproteobacteria*), and *Massilia* spp. (1 isolate, *Gammaproteobacteria*). *Acidovorax-* and *Rhodococcus-*related isolates were obtained only during the first isolation procedure and only from the aerobic enrichment. *Achromobacter-*affiliated isolates were obtained only from the oxygen-limited enrichment.

**Table 1:** Taxonomic affiliation and naphthalene biodegradation potential of biofilm isolates obtained during the study

Strain designation*	16S rDNA accession numbers	Isolation source/type of enrichment	Nearest cultured neighbor upon 16S rRNA	Phylogenetic affiliation	bp	16S rRNA sequence homology (%)	Naphthalene biodegradation as assessed by GC-MS (1 mg l^−1^)	Presence of NDO-related 2Fe-2S genes
R1.1_4	MN197554	Aerobic 1st week	*Pseudomonas silesiensis* A3(T)	*Gammaproteobacteria*	1422	99.7	+	+
R1.1_5	MN197555	Aerobic 1st week	*Acidovorax delafieldii* DSM 64(T)	^*¥*^*Gammaproteobacteria*	1413	99.7	+	+
R1.1_7	MN197556	Aerobic 1st week	*Pseudomonas arsenicoxydans* CECT 7543(T)	*Gammaproteobacteria*	1440	99.2	+	+
RN1.1_1	MN197557	Aerobic 1st week	*Pseudomonas silesiensis* A3(T)	*Gammaproteobacteria*	1442	99.6	−	−
RN1.1_3 uj	MN197558	Aerobic 1st week	*Pseudomonas silesiensis* A(T)	*Gammaproteobacteria*	1431	99.9	+	+
RN1.1_4F	MN197559	Aerobic 1st week	*Rhodococcus jostii* DSM 44719(T)	*Actinobacteria*	1416	99.9	+	−
RN1.1_5	MN197560	Aerobic 1st week	*Pseudomonas silesiensis* A(T)	*Gammaproteobacteria*	1475	99.3	+	+
RN1.1_7	MN197561	Aerobic 1st week	*Massilia aurea* AP13(T)	^*¥*^*Gammaproteobacteria*	1358	99.0	−	−
BGN1.1_2	MN197562	Aerobic 1st week	*Acidovorax facilis* CCUG 2113(T)	^*¥*^*Gammaproteobacteria*	1355	99.6	−	−
BGN1.1_3	MN197563	Aerobic 1st week	*Acidovorax delafieldii* DSM 64(T)	^*¥*^*Gammaproteobacteria*	1364	99.5	+	−
BGN1.1_4	MN197564	Aerobic 1st week	*Acidovorax delafieldii* DSM 64(T)	^*¥*^*Gammaproteobacteria*	1432	99.9	+	−
BGN1.1_5	MN197565	Aerobic 1st week	*Rhodococcus jostii* DSM 44719(T)	*Actinobacteria*	1407	99.6	+	−
BGN1.1_6	MN197566	Aerobic 1st week	*Pseudomonas mandelii* NBRC 103147(T)	*Gammaproteobacteria*	1355	99.7	+	+
BGN1.1_8	MN197567	Aerobic 1st week	*Rhodococcus jostii* DSM 44719(T)	*Actinobacteria*	1393	99.8	+	−
R3.6_1	MN197568	Aerobic 6e week	*Pseudomonas umsongensis* DSM 16611(T)	*Gammaproteobacteria*	1430	99.7	−	−
R3.6_2	MN197569	Aerobic 6th week	*Pseudomonas umsongensis* DSM 16611(T)	*Gammaproteobacteria*	1438	99.5	+	+
R3.6_3	MN197570	Aerobic 6th week	*Pseudomonas laurylsulfatiphila* AP3_16(T)	*Gammaproteobacteria*	1376	99.4	+	+
R3.6_4	MN197571	Aerobic 6th week	*Pseudomonas laurentiana* GSL-010(T)	*Gammaproteobacteria*	1439	99.6	+	+
RN3.6_1	MN197572	Aerobic 6th week	*Pseudomonas umsongensis* DSM 16611(T)	*Gammaproteobacteria*	1446	99.0	−	−
RN3.6_3	MN197573	Aerobic 6th week	*Pseudomonas laurentiana* GSL-010(T)	*Gammaproteobacteria*	1436	99.2	+	+
RN3.6_4	MN197574	Aerobic 6th week	*Pseudomonas silesiensis* A3(T)	*Gammaproteobacteria*	1425	99.8	−	−
BGN3.6_1	MN197575	Aerobic 6th week	*Pseudomonas umsongensis* DSM 16611(T)	*Gammaproteobacteria*	1425	99.9	−	−
BGN3.6_2	MN197576	Aerobic 6th week	*Pseudomonas reidholzensis* CCOS 865(T)	*Gammaproteobacteria*	1437	99.5	−	−
BGN3.6_3	MN197577	Aerobic 6th week	*Pseudomonas frederiksbergensis* JAJ28(T)	*Gammaproteobacteria*	1434	99.5	+	+
BGN3.6_4	MN197578	Aerobic 6th week	*Pseudomonas umsongensis* DSM 16611(T)	*Gammaproteobacteria*	1438	99.9	+	+
BGN3.6_5	MN197579	Aerobic 6th week	*Pseudomonas laurentiana* GSL-010(T)	*Gammaproteobacteria*	1352	99.3	+	+
BGN3.6_6	MN197580	Aerobic 6th week	*Pseudomonas frederiksbergensis* JAJ28(T)	*Gammaproteobacteria*	1441	99.5	+	+
HR1.1_1	MN197581	Oxygen-limited 2nd week	*Pseudomonas veronii* DSM11331(T)	*Gammaproteobacteria*	1433	99.8	+	+
HR1.1_2	MN197582	Oxygen-limited 2nd week	*Pseudomonas laurentiana* GSL-010(T)	*Gammaproteobacteria*	1432	99.6	+	+
HR1.1_5	MN197583	Oxygen-limited 2nd week	*Pseudomonas veronii* DSM11331(T)	*Gammaproteobacteria*	1384	99.8	+	+
HBGN1.1_3	MN197584	Oxygen-limited 2nd week	*Pseudomonas laurentiana* GSL-010(T)	*Gammaproteobacteria*	1429	99.6	+	+
HBGN1.1_4	MN197585	Oxygen-limited 2nd week	*Achromobacter anxifer* LMG 26857	^*¥*^*Gammaproteobacteria*	1420	99.9	−	−
HR3.3_1	MN197586	Oxygen-limited 6th week	*Pseudomonas veronii* DSM11331(T)	*Gammaproteobacteria*	1392	99.6	+	+
HR3.3_3	MN197587	Oxygen-limited 6th week	*Pseudomonas marginalis* ATCC 10844(T)	*Gammaproteobacteria*	1360	99.3	+	+
HR3.3_4	MN197588	Oxygen-limited 6th week	*Pseudomonas marginalis* ATCC 10844(T)	*Gammaproteobacteria*	1447	98.7	+	+
HR3.3_5	MN197589	Oxygen-limited 6th week	*Achromobacter spanius* LMG 5911(T)	^*¥*^*Gammaproteobacteria*	1430	99.7	−	−
HRN3.3_1	MN197590	Oxygen-limited 6th week	*Pseudomonas veronii* DSM11331(T)	*Gammaproteobacteria*	1441	99.6	+	+
HRN3.3_3	MN197591	Oxygen-limited 6th week	*Pseudomonas veronii* DSM11331(T)	*Gammaproteobacteria*	1391	99.4	+	+
HRN3.3_4	MN197592	Oxygen-limited 6th week	*Pseudomonas veronii* DSM11331(T)	*Gammaproteobacteria*	1440	99.8	+	+
HBGN3.3_1	MN197593	Oxygen-limited 6th week	*Pseudomonas veronii* DSM11331(T)	*Gammaproteobacteria*	1438	99.6	+	+

*R—direct plating on R2A agar; *RN*—direct plating on R2A agar, onto which 100 μl of naphthalene suspension had been previously plated (10 mg/mL); *BGN*—direct plating on BBH medium solidified with gellan gum, onto which 100 μl of naphthalene suspension had been previously plated (10 mg/mL); *HR*, *HRN*, *HBGN*—isolates originating from the oxygen-limited enrichments; “+”—designates either naphthalene degradation as assessed by GC-MS or the possession of naphthalene-dioxygenase reductase component involved in naphthalene biodegradation as detected through CODEHOP PCR

^¥^Formerly classified as *Betaproteobacteria*, which now can be found within *Gammaproteobacteria* in SILVA as order *Betaproteobacteriales*, Parks et al. 2018

It was found that ~ 90% of the isolated strains belonged to the group of Gram-negative bacteria. Only three isolates, *Rhodococcus* spp., belonged to the Gram-positive group (7.5%) (Table 1).

### Bacterial community dynamics assessed by cultivation-independent approach

#### Temporal dynamics during the enrichment as revealed by T-RFLP

**Fig. 1:**
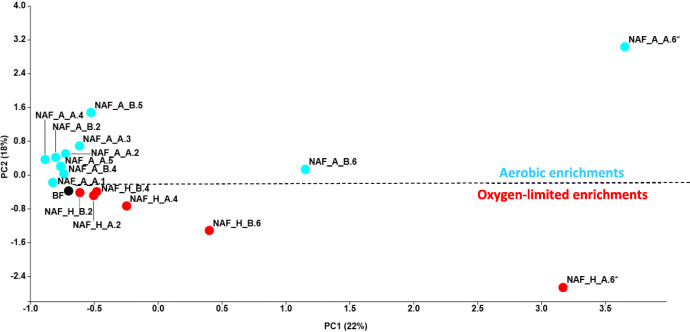
PCA clustering of the aerobic and oxygen-limited naphthalene-amended biofilm enrichments on the basis of *16S rRNA* gene–based T-RFLP electropherograms. (BF, initial biofilm (black spot); NAF_A, naphthalene-amended aerobic enrichments (blue spot); NAF_H, naphthalene-amended oxygen-limited enrichments (red spot); A/B replicates; numbers 1–6, number of weeks of enrichment; *, enrichment cultures selected for Illumina *16S rRNA* amplicon sequencing together with the initial sample BF. Please note that in the case of aerobic enrichments of the first and third week, we were able to isolate the community DNA only from one replicate (NAF_A_A.1 and NAF_A_A.3, respectively)

The PCA based on *16S rRNA* gene T-RFLP patterns showed a clear separation of aerobic and oxygen-limited naphthalene-degrading biofilm bacterial communities throughout the enrichment period. The bacterial communities were separated along axis 1 according to time (Fig. 1), while axis 2 separated the bacterial communities according to the oxygenic conditions. The highest differences were recorded between samples of the last week (NAF_A_B.6 vs. NAF_H_B.6 or NAF_A_A.6 vs. NAF_H_A.6). In order to characterize the effect of oxygenic conditions on the bacterial community, the composition of bacterial communities—assessed by Illumina *16S rRNA* gene amplicon sequencing—obtained under the aerobic enrichment at the end of the enrichment period (NAF_A_A.6) was compared to that of oxygen-limited conditions obtained at the same time (NAF_H_A.6). Additionally, the bacterial community composition of both NAF_A_A.6 and NAF_H_A.6 was compared with that of the initial biofilm community (BF).

#### Bacterial diversity according to the Illumina *16S rRNA* gene amplicon sequencing

The Illumina *16S rRNA* gene amplicon sequencing provided 63 828 reads for the initial biofilm sample (BF), 16 683 for the aerobic enrichment (NAF_A_A.6), and 59 197 for the oxygen-limited enrichment (NAF_H_A.6) samples. The rarefaction curves of the three samples indicated that the data contained enough sequence depth to ascertain the full bacterial diversity. High sequencing coverage was reached in all the three samples (Supplementary Fig. S1).

**Fig. 2:**
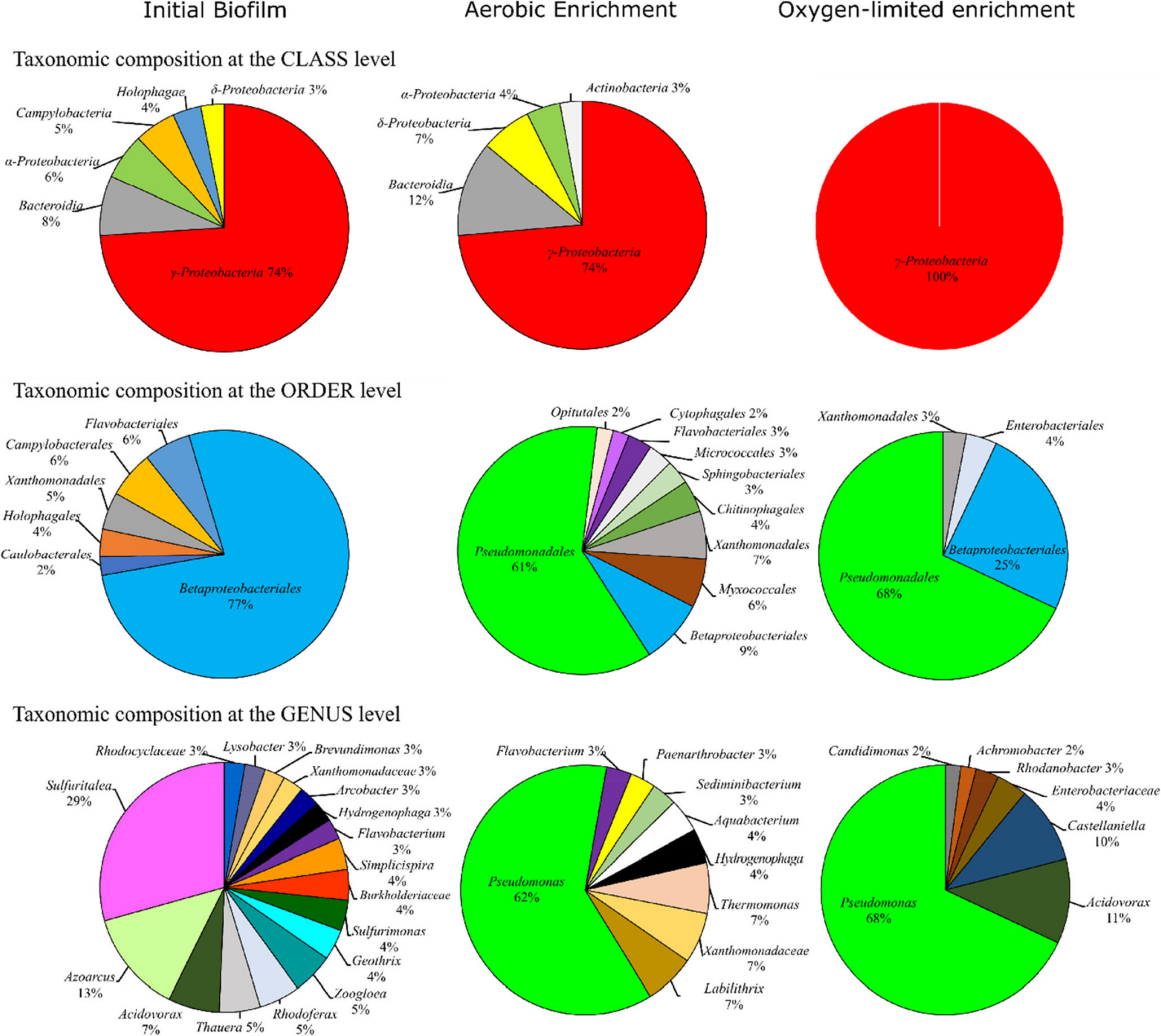
Bacterial diversity of the initial biofilm sample (BF), as well as of aerobic (sample NAF_A_A.6) and oxygen-limited enrichments at the last week (NAF_H_A.6) assessed by Illumina *16S rRNA* gene amplicon sequencing. Community members with a relative abundance ≥ 2% are shown

At the class level, all investigated samples were dominated by *Gammaproteobacteria* (including the former *Betaproteobacteria* class, which is now included within the *Gammaproteobacteria* in SILVA as *Betaproteobacteriales*, Parks et al. 2018) followed by *Bacteroidia*. At the order level, *Betaproteobacteriales* were the most dominant in the initial biofilm community, while in the aerobic and oxygen-limited enrichments *Pseudomonadales* were the most abundant. Throughout the enrichment period, the relative abundance of *Betaproteobacteriales* decreased remarkably to 9% (aerobic) and 25% (oxygen-limited). At the genus level, the initial biofilm was inhabited by the following well-known, strictly aerobic or facultative anaerobic chemolithotrophic or chemoorganotrophic bacteria: *Sulfuritalea*, *Azoarcus*, *Acidovorax*, *Thauera*, *Rhodoferax*, *Zoogloea*, *Geothrix*, etc. The genus *Pseudomonas* showed the highest relative abundance in the enrichment cultures of the 6th week; however, *Pseudomonas*-related sequences were found to be very low in the initial biofilm (0.3%). In the aerobic enrichment, the next most abundant sequences belonged to unclassified bacteria affiliating with *Xanthomonadaceae* and *Labilithrix*. In the oxygen-limited enrichment, *Acidovorax* and *Castellaniella* were the next most dominant genera. Interestingly, while at species level the aerobic enrichment of the last week was dominated by *Pseudomonas laurentiana* (57% relative abundance), *Pseudomonas veronii/extremaustralis* lineage affiliating bacteria dominated the oxygen-limited setup (64%) (Supplementary Table S1, Fig. 2).

#### Bacterial community shifts in response to different enrichment conditions

A clear shift in the biofilm bacterial community composition was observed during the course of the enrichment. For instance, while at the class level *Holophagae* and *Campylobacteria* were abundant in the starting community, their relative abundance decreased to non-detectable during enrichment. *Deltaproteobacteria*, *Alphaproteobacteria*, and *Bacteroidia* were in notable amounts only in the initial biofilm and in the aerobic enrichment. *Gammaproteobacteria* were the most dominant in the oxygen-limited setup. At the order level, the most striking shift was observed in the case of *Pseudomonadales*: very low abundance in the initial sample and higher than 60% relative abundance after the enrichments. In contrast, while *Betaproteobacteriales* were dominant in the initial biofilm, their relative abundance remarkably decreased during the enrichment, reaching the lowest value in the aerobic setting. At the genus level, bacteria belonging to the genera *Arcobacter*, *Simplicispira*, *Sulfurimonas*, *Geothrix*, *Zoogloea*, *Rhodoferax*, *Thauera*, *Azoarcus*, and *Sulfuritalea* showed the highest relative abundance only in the starting biofilm. During the selective enrichments, their relative abundance decreased close to or under the detection limit in both enrichments. *Hydrogenophaga* and *Flavobacterium* were present only in the initial biofilm and in the aerobic enrichment. Representatives of the genera *Thermomonas*, *Labilithrix, Paenarthrobacter*, *Sediminibacterium*, and *Aquabacterium* were in considerable proportions only in the aerobic enrichment. Besides *Pseudomonas* spp., in the oxygen-limited enrichment, the most dominant bacteria belonged to the genera *Acidovorax*, *Castellaniella*, *Rhodanobacter*, and *Achromobacter.*

Overall, the culture-independent data was consistent with the results of the cultivation-dependent approach showing that Gram-negative bacteria, mainly belonging to the genus *Pseudomonas*, dominated the enrichments.

### PCR detection and diversity of genes involved in naphthalene biodegradation

#### CODEHOP primer pair design for the detection of NDO

**Fig. 3:**
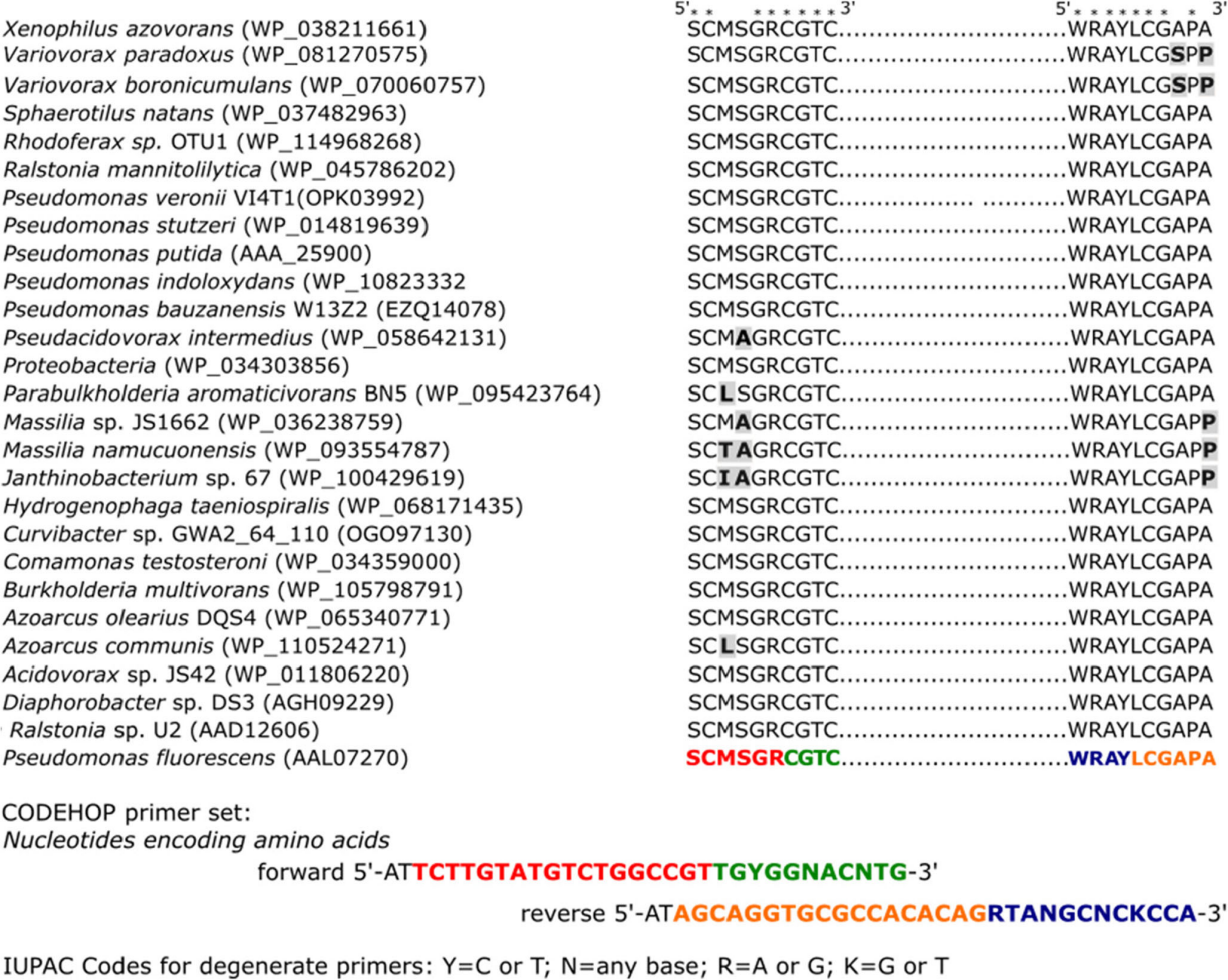
Alignment of naphthalene 1,2-dioxygenase-related 2Fe-2S reductase component protein sequences involved in the biodegradation of PAH compounds. Sequences have been retrieved from the GenBank (NCBI). GenBank accession numbers are given in parentheses. Amino acids marked with an asterisk have consensus through the investigated sequences. Variations in the target sequence are shown with gray shading and in bold letters. Sequence sections shown in green and blue represent the 3′ degenerate core of the forward and reverse primers, respectively. Red and orange colors represent the 5′ consensus clamps

By both cultivation-dependent and cultivation-independent methods, it has been found that the naphthalene (potentially PAH)-degrading community members of the investigated biofilm predominantly belonged to the group of Gram-negative bacteria. Therefore, the CODEHOP primers have been designed on amino acid sequences of naphthalene 1,2-dioxygenase-related 2Fe-2S reductase proteins found in Gram-negative bacteria (Fig. 3).

By aligning the protein sequences, a highly conserved N-terminal motif of 4 amino acids was identified (“CGTC”). A second C-terminal conserved motif, 246 amino acids downstream of the “CGTC,” was also identified (“WRAY”). To design the consensus clamps for each strand, “sense” and “anti-sense,” the most common 6 amino acids were determined upstream and downstream of the target motifs, respectively. The most frequently used codon for each of these amino acids was determined. The obtained forward (CGTC-F 32x) and reverse (WRAY-R 64x) CODEHOP primers were 5′-ATTCTTGTATGTCTGGCCGTtgyggnacntg-3′ and 5′-ATAGCAGGTGCGCCACACAGrtangcnckcca-3′, respectively. Lower case characters denote the 3′ degenerate core of the primers.

#### The diversity of NDO-related 2Fe-2S reductase component genes

First, by using the newly designed primers, a PCR reaction was conducted on the community DNA of the initial biofilm and the enrichment cultures. PCR amplicons of the desired size were detected only in the case of enrichments. In the case of the initial biofilm sample, no amplicons were obtained during the PCR, even though the template DNA concentration was increased up to 150 ng. However, by using high DNA concentration, the PCR amplification of *16S rRNA* genes was successful.

From the aerobic and oxygen-limited enrichments, 48 and 44 clones were sequenced, respectively. The obtained clone sequences were grouped into four operational protein units (OPUs) using at least 98% protein sequence similarity. From the aerobic enrichment, OPU 1, OPU 2, and OPU 3 accounted for 87.5%, 8.3%, and 4.2% of total clone sequences, respectively. OPU 4 accounted for 100% of the total clone sequences originating from the oxygen-limited enrichment. OPU 1 and OPU 4 showed the closest amino acid sequence similarity to *Pseudomonas* species encoded NDO-related 2Fe-2S reductase component genes (e.g., *P. fluorescens* PC20, *P. putida*, and *P. frederiksbergensis* AS1; amino acid sequence similarities ranged from 98.7 to 100%). OPU 2 showed the closest similarity to the putatively NDO-related 2Fe-2S reductase component gene of *Malikia spinosa* strain AB6 (amino acid sequence homologies ranged from 99.2 to 100%). OPU 3 showed the closest amino acid sequence homologies either to oxidoreductase component of 2,4-DNT dioxygenase of *Burkholderia cepacia* (99.2% amino acid sequence homology) or to ferredoxin reductase component of *Ralstonia* sp. U2 (99.2%).

Amino acid sequence homologies between OPU 1 and OPU 2 ranged from 65.8 to 67.4%. In the case of OPU 1 and OPU 3, homologies ranged from 66.9 to 67.5%. Irrespective of the origin, the vast majority of the NDO-related 2Fe-2S reductase component clone sequences showed the closest homology with *nahAa* proteins of *Pseudomonas* spp. (aerobic enrichment 87.5% of total clone sequences, oxygen-limited enrichment 100%).

**Fig. 4:**
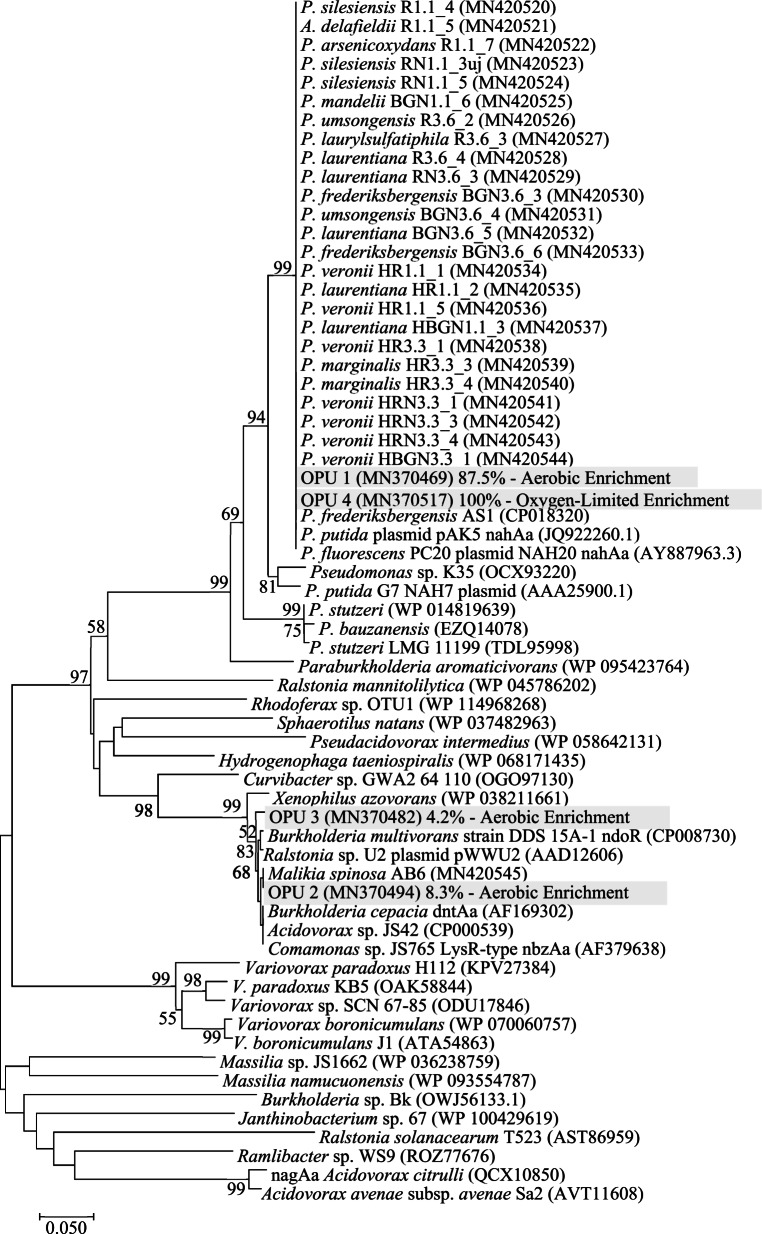
Phylogenetic analysis of NDO-related 2Fe-2S reductase protein sequences using the Neighbor-Joining method (Saitou and Nei 1987). The percentage of replicate trees in which the associated OPUs clustered together in the bootstrap test (1000 replicates) is shown next to the branches (Felsenstein 1985). The tree is drawn to scale, with branch lengths in the same units as those of the evolutionary distances used to infer the phylogenetic tree. The evolutionary distances were computed using the Poisson correction method (Zuckerkandl and Pauling 1965) and are in the units of the number of amino acid substitutions per site. The analysis involved 66 amino acid sequences. All positions containing gaps and missing data were eliminated. There were a total of 222 positions in the final dataset. Evolutionary analyses were conducted in MEGA7 (Kumar et al. 2016)

The majority of the isolated strains (24 *Pseudomonas* sp. isolates and 1 *Acidovorax* sp. isolate, 60%) harbored NDO-related 2Fe-2S reductase component genes involved in naphthalene biodegradation (Table 1, Fig. 4, Supplementary Fig. S2). Amino acid sequences of the amplified gene regions showed 100% amino acid sequence homology with *Pseudomonas* species encoded NDO-related 2Fe-2S reductase proteins (Fig. 4). By using the designed CODEHOP primers, NDO-related genes were not detected in the case of *Rhodococcus*, *Massilia*, and *Achromobacter* strains nor in the majority of the *Acidovorax-*related isolates. In the case of *M. spinosa* AB6, the PCR positive control, a 750-bp amplicon was detected, (Supplementary Fig. S2, Fig. 4). Non-specific PCR products were not detected in the case of the community nor in the case of genomic DNA samples.

### Naphthalene biodegradation potential of isolated strains

During the microcosm experiments, 75% of the isolates (30 isolates) were able to degrade naphthalene (1 mg l^−1^) (Table 1). Isolates showing positive biodegradation capacities were able to quickly degrade the tested PAH compound within 20 h (the incubation time set for the microcosm experiments). Among the 30 *Pseudomonas-*related isolates, 24 were able to degrade naphthalene. All *P. veronii*, *P. marginalis*, and *P. laurentiana* strains showed naphthalene biodegradation ability. This was not always true for *P. silesiensis–* and *P. umsongensis*–affiliated isolates. All *Rhodococcus* spp.– and *A. delafieldii*–related isolates degraded naphthalene. *M. aurea–*, *A. facilis–*, *P. reidholzensis–*, and *Achromobacter*-related isolates were not able to degrade naphthalene.

It was observed that all isolates—except *R. jostii* RN1.1_4F, BGN1.1_5, and BGN1.1_8, and *A. delafieldii* BGN1.1_3 and BGN1.1_4—that degraded naphthalene also possessed naphthalene dioxygenase. It was also found that while some strains belonging to a certain bacterial species exhibited naphthalene biodegradation ability, others, belonging to the same species, did not (*P. silesiensis* R1.1_4, RN1.1_3_uj and RN1.1_5 versus *P. silesiensis* RN1.1_1 and RN3.6_4; *P. umsongensis* R3.6_2 and BGN3.6_4 versus *P. umsongensis* R3.6_1, RN3.6_1 and BGN3.6_1). Surprisingly, not only isolates possessing the NDO gene were able to degrade naphthalene, but also some of the isolates that did not carry this gene (at least as assessed by the newly developed primers) were able to degrade naphthalene as well.

In the case of bacterial strains, which genomic DNA was used as negative control during the PCR amplification of the targeted functional gene, no naphthalene biodegradation was observed (*Zoogloea oleivorans* BUC-1^T^, *Rhodococcus pyridinivorans* AK37, and *Cupriavidus basilensis* OR16). However, after 60 h of incubation, the positive control isolate *M. spinosa* AB6 was able to degrade naphthalene, although at a much slower rate.

### Biofilm-producing potential of isolates

**Fig. 5:**
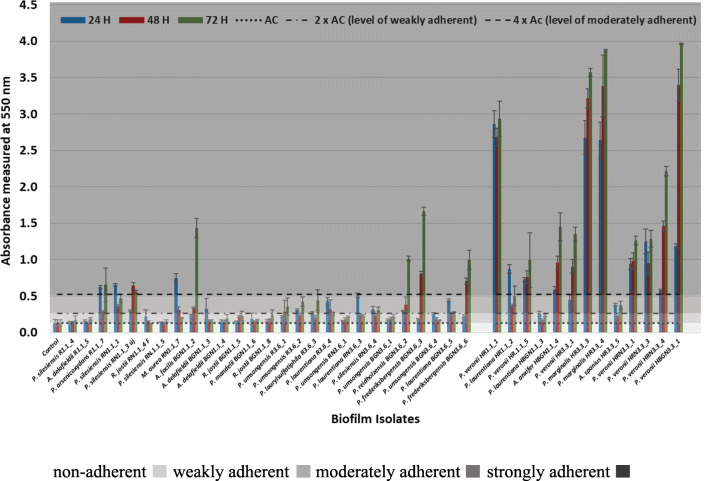
Biofilm-forming potential of isolates based on crystal-violet absorbance assay by using 96-well, cell culture chimney well polystyrene microplates

The majority of isolates was moderately or strongly adherent to the polystyrene wall of the microplate (moderately adherent 8 isolates, 20%; strongly adherent 19 isolates, 47.5%). Isolates HR1.1_1, HR3.3_3, HR3.3_4, and HBGN3.3_1 (10% of total isolates) can be viewed as extremely adherent, because after 72 h of incubation their adherence 5–7 times exceeded the level of moderately adherent. These extreme biofilm-producing organisms showed the closest similarity to species *P. veronii* and *P. marginalis*. Almost 10% of isolates proved to be non-adherent and 22.5% weakly adherent. *A. delafieldii–* and *Rhodococcus*-related isolates did not form biofilms on the tested surface (Fig. 5, Supplementary Table S2).

## Discussions

PAHs are extremely toxic compounds (ATSDR 1995). Microorganisms play a crucial role in determining their fate in the environment (Duran and Cravo-Laureau 2016). From a bioremediation perspective, it is important to know which bacteria play a role in the biodegradation of these compounds under different environmental conditions.

In this study, we aimed at enrichment, identification, and isolation of naphthalene (presumably PAH)-degrading bacteria from a bacterial biofilm developed in a hydrocarbon-contaminated subsurface, freshwater ecosystem. Full aerobic and oxygen-limited selective enrichment cultures were initiated in mineral salt solution amended with high concentrations of naphthalene as sole source of energy and carbon. Oxygen-limited enrichment cultures were set up in order to mimic conditions close to in situ, namely low-oxygen availability in hydrocarbon-contaminated groundwater.

During the early stages of the study, it became evident that the investigated biofilm harbors naphthalene (presumably PAH)-degrading community members. After 1 or 2 weeks of incubation, the naphthalene crystals disappeared completely from the hermetically closed bottles. The progressive consumption of naphthalene crystals in enrichment cultures was not unique. Disappearance of naphthalene crystals in a microaerophilic, naphthalene-amended enrichment culture was also observed by Martirani-Von Abercron et al. (2017). Surprisingly, naphthalene 1,2-dioxygenase reductase (NDO) genes for Gram-negative bacteria were not detected by PCR using the newly designed CODEHOP primers in the starting biofilm community, but only after the first or second week of enrichment incubations. Such observation suggested that the NDO gene copy number initially was low, under the PCR detection limit. However, the number of degrading organisms and the naphthalene biodegradation ability increased throughout the enrichment period. These observations suggested that, although, until the biofilm sampling, the majority of the hydrocarbon pollutants in the groundwater had been removed, the hydrocarbon, possible PAH degradation capacity of the biofilm community, remained. The swift propagation of the hidden PAH degradation capacity of the biofilm community was triggered by the presence of naphthalene.

The CODEHOP approach is a powerful tool for the detection and identification of novel and/or distantly related genes. It has been used to identify and characterize new gene orthologs and paralogs in different plant, animal, bacterial, and virus species (Rose et al. 2003; Rose 2005; Staheli et al. 2011).

The vast majority of the NDO-related 2Fe-2S reductase component gene clone sequences, originating from either the aerobic or oxygen-limited enrichments, affiliated with *Pseudomonas* spp. encoded genes (Fig. 4). This observation indicated a possible horizontal gene transfer (HGT) event allowing the propagation of the naphthalene degradation ability within the bacterial community. The supposed HGT was consistent with the fact that bacterial isolates not related to *Pseudomonas*, such as *Acidovorax* species, harbored the same NDO gene. The random occurrence of the NDO gene and also of naphthalene biodegradation ability in the case of different strains, affiliating with the same bacterial species (e.g., *A. delafieldii, P. silesiensis*, and *P. umsongensis* strains), further indicated a horizontal gene transfer event within the community. Due to HGT, some strains acquired the NDO gene and the naphthalene degradation ability while others, belonging to the same species, did not. These observations were in accordance with previous reports showing that PAH-degrading genes are often found on mobile genetic elements (e.g., plasmid NAH7) allowing their dispersion in the bacterial community through HGT (Obayori and Salam 2010; Ghosal et al. 2016). For example, it has been demonstrated that *Pseudomonas* spp. possess conjugative plasmids carrying catabolic operons located in a transposon structure allowing their spread within microbial communities (Herrick et al. 1997; Leahy and Colwell 1990). Moreover, such HGT has been found to be favored in biofilm organizations (Molin and Tolker-Nielsen 2003); the close proximity of bacterial cells facilitates cell-to-cell interactions and thus DNA exchange (Kostakioti et al. 2013).

Interestingly, the initial biofilm investigated in this study was phylogenetically similar to the biofilm from the same site described in a previous sampling campaign (March 2016, Benedek et al. 2018) suggesting that the biofilm was stable over the time. Both biofilm samples, originating from the two different sampling campaigns, were dominated by *Beta*- (now *Gamma*-, Parks et al. 2018), *Alpha*-, and *Deltaproteobacteria* (Fig. 2, Supplementary Table S1). At the genus level, *Sulfuritalea*, *Azoarcus*, *Acidovorax*, *Thauera*, *Zoogloea*, and *Flavobacterium* were amongst the most abundant genera in both cases. However, the selective enrichment, using either simple aromatic (BTEX, March 2016) or polycyclic aromatic hydrocarbons (naphthalene, January 2018) as the sole carbon source, resulted in different bacterial communities. For both studies, a temporal shift during the enrichment was observed. Both BTEX and naphthalene behaved as strong selectors, which exerted a selective pressure on the bacterial community. Although in the presence of BTEX *Malikia* (aerobic) and *Acidovorax* (oxygen-limited enrichment) species became dominant (first biofilm sampling, Benedek et al. 2018), in naphthalene-amended enrichments, *Pseudomonas* species dominated both enrichment types (second sampling, this study).

The remarkable presence of *Pseudomonas* species in the naphthalene enrichments is not in accordance with findings of Martirani-Von Abercron et al. (2017); they found the dominance of *Variovorax* spp. (54%) and *Starkeya* spp. (43%) in microaerophilic naphthalene enrichments. Surprisingly, the presence of *Pseudomonas* spp. in their enrichment cultures was marginal. Moreover, *Pseudomonas* isolates obtained during their study were barely able of naphthalene biodegradation. In contrast, Ma et al. (2006) observed the dominance of *Pseudomonas* spp. during the enrichment and isolation of naphthalene- and phenanthrene-degrading bacteria from Antarctic soils*.* In a petroleum-contaminated soil, where the main PAH pollutant was naphthalene, Alquati et al. (2005) also revealed the presence of *Pseudomonas* spp. Moreover, in one of our previous studies, in the case of a highly PAH-contaminated soil, the dominance of *Pseudomonas* spp. was observed again (~ 40% relative abundance, Benedek et al. 2013).

The high incidence of *Pseudomonas* species in naphthalene enrichments, as well as their PAH biodegradation ability, has been widely reported (Amini et al. 2017; Nwinyi et al. 2016; Thomas et al. 2016; Patowary et al. 2015; Wald et al. 2015; Ma et al. 2012; Obayori et al. 2008; Ma et al. 2006; Leahy and Colwell 1990; Foght and Westlake 1988). However, this is the first study reporting the naphthalene biodegradation ability of *P. silesiensis*, *P. arsenicoxydans*, *P. umsongensis*, *P. laurylsulfatiphila*, *P. mandelii*, and *P. laurentiana*; so far, no other PAH compound degradation ability of isolates affiliated with these species has been reported either. In addition, to the best of our knowledge, no information can be found in the literature regarding petroleum hydrocarbon degradation ability of *P. silesiensis–*, *P. laurylsulfatiphila–*, and *P. laurentiana*–related pure isolates*.* On the other hand, petroleum hydrocarbon degradation ability of *P. arsenicoxydans* and *P. umsongensis* pure isolates is already known (Pham et al. 2014).

According to the whole genome sequences, type strains of *P. silesiensis* A3^T^, *P. umsongensis* DSM16611^T^ and *P. laurylsulfatiphila* AP3_16^T^, as well as *P. arsenicoxydans* strains ACM1 and E3, do not contain in their genome naphthalene 1,2-dioxygenase-related genes (Kaminski et al. 2018; Furmanczyk et al. 2018; Kwon et al. 2003; Altshuler et al. 2019). This observation further indicated that isolates obtained in this study, affiliating with the abovementioned bacterial species, acquired the naphthalene biodegradation capability through HGT.

The obtained results indicated that the aerobic enrichment was dominated by *P. laurentiana*, while the oxygen-limited enrichment was dominated by bacteria affiliating with the *P. extremaustralis/P. veronii* lineage. The type strain GSL-010^T^ of *P. laurentiana* was first isolated by Wright et al. (2019) as a Mn(III)-oxidizing bacteria from the St. Lawrence Estuary. As stated above, no information can be found regarding petroleum hydrocarbon degradation ability of *P. laurentiana* pure cultures. In contrast, a series of studies discuss the hydrocarbon degradation ability (including both aliphatic- and aromatic) of *P. extremaustralis*/*P. veronii* lineage affiliating bacteria (Tribelli et al. 2012, 2018; Imperato et al. 2019; Morales et al. 2016; Wald et al. 2015). *P. extremaustralis* 14-3^T^ was isolated, for the first time, from an Antarctic environment and was able to tolerate and degrade hydrocarbons. It can be used in extreme (cold) environments for hydrocarbon bioremediation. According to its whole genome sequence (VFET00000000.1), it harbors alkane monooxygenase (*alkB*), catechol 1,2-dioxygenase (*C12O*) and catechol 2,3-dioxygenase (*C23O*), genes involved in either “*orto*” or “*meta*” cleavage of aromatic compounds. However, no genes involved in naphthalene biodegradation have been identified in its genome. Genomic analysis of *P. veronii* VI4T1 (NZ_MULN00000000.1) indicated the presence of the full naphthalene dioxygenase operon and also genes involved in the degradation of BTEX compounds and alkanes (*alkB* gene). In addition, another isolate *Pseudomonas veronii* strain 20a2, obtained from a PAH-contaminated lake sediment, was able to degrade a series of PAHs as indicated in Table 2 (Wald et al. 2015). The fact that *P. extremaustralis/P. veronii* affiliating bacteria were the most dominant in the oxygen-limited enrichment is not a coincidence. According to Tribelli et al. (2018), *P. extremaustralis* has a high affinity towards microaerobic hydrocarbon degradation. It was found that *P. extremaustralis* was able to grow under microaerobic conditions using diesel as sole carbon and energy source. Interestingly, under full aerobic conditions, no hydrocarbon degradation occurred (Tribelli et al. 2018). Moreover, Révész et al. (2019) also found the dominance of *P. extremaustralis/P. veronii* lineage–related bacteria in diesel fuel/crude oil mixture–amended enrichment cultures. They concluded that *P. extremaustralis*/*P. veronii* lineage affiliating bacteria are adapted to microaerobic conditions and may have an important role in alkane degradation in subsurface ecosystems. The ability to use petroleum hydrocarbons under low-oxygen conditions presents an adaptive/evolutionary advantage for this bacteria.

**Table 2:** PAH degradation ability of bacterial species which showed naphthalene biodegradation capability in this study

Species	Isolate	Source of isolation/type of selective enrichment	PAH degradation^b^	Reference
*Acidovorax delafieldii*	P4-1	–^a^	FLU, FLN, PYR, PHE	Samanta et al. 1999
	TNA921	Creosote-contaminated soil/PHE-amended MSM ^c^	PHE	Shuttleworth and Cerniglia 1996
	NA3	PAH-contaminated soil/PHE-amended MSM	NAP, PHE, CHR, B[a]ANT, B[a]PYR	Singleton et al. 2009
*Rhodococcus jostii*	016	Chronically hydrocarbon-contaminated soil/-	NAP, and PHE and PYR co-metabolically with glucose	Bourguignon et al. 2014
*Pseudomonas veronii*	SFI 3	Lagoon sediment sample/FLN-amended MSM	FLN, PHE; PYR	Ben Said et al. 2007
	20a2	PAH-contaminated lake sediment sample/NAP-amended MSM	NAP, ACE, FLU, PHE, ANT, FLN, PYR	Wald et al. 2015
*Pseudomonas frederiksbergensis*	JAJ28	Tar-polluted soil/PHE-coated minimal agar medium	PHE	Andersen et al. 2000
	AS1	Arsenic-contaminated site/NAP-amended MSM	NAP	Yoon-Suk et al. 2006
*Pseudomonas marginalis*	PD-14B	PAH-contaminated soil/PHE-coated agar	ANT, ACY, NAP, CHR	Burd and Ward 1996

^a^Not known

^b^*NAP* naphthalene, *FLU* fluorene, *FLN* fluoranthene, *PYR* pyrene, *PHE* phenanthrene, *ANT* anthracene, *CHR* chrysene, *B[a]ANT* benz[a]anthracene, *B[a]PYR* benzo[a]pyrene, *ACE* acenaphthene, *ACY* acenaphthylene

^c^*MSM* mineral salt medium

Based on the abovementioned, it is evident that *Pseudomonas* spp. are good PAH-degrading organisms. The presence of this bacterial species may indicate an elevated natural attenuation capacity of a PAH-contaminated site. Most probably *P. laurentiana* is capable of aerobic PAH degradation while *P. extremaustralis/P. veronii* lineage affiliating bacteria are efficient in oxygen-limited PAH degradation.

The overwhelming dominance of *Pseudomonas* species in the selective enrichments may be explained with the theory of r/K selection. r-strategists (e.g., all *Gammaproteobacteria* including *Pseudomonas* spp.) undergo rapid changes when an environment is perturbed and can respond with large population increases. K-strategists dominate in stable non-perturbed environments (De Lei et al. 1995; Brzeszcz et al. 2016). Being r-strategists, the high adaptability to the changing environment and fast reproduction rate made *Pseudomonas* spp. the most dominant in the naphthalene-amended selective enrichments; *pseudomonads* could grow without hindrance. Related to the aforementioned, the r/K selection may have been responsible also for the dominance of Gram-negative bacteria over Gram-positives, as it has been proven by both cultivation-dependent and cultivation-independent phylogenetic studies, although Gram-positive bacteria are known to have great potential for the biotransformation and biodegradation of organic compounds (Larkin et al. 2005) when detected are never dominant (Kaplan and Kitts 2004). This may be explained by the fact that Gram-positive bacteria broadly represent K-strategists or oligotrophic organisms with slower growth rates than Gram-negatives (de Vries and Shade 2013; de Vries and Griffiths 2018).

Besides *Pseudomonas* spp., isolates affiliating with the genera *Acidovorax* and *Castellaniella* also deserved attention. In this study, *A. defluvii* and *C. caenii* were found to be in considerable amounts in naphthalene-amended oxygen-limited enrichment (Supplementary Table S1). Therefore, the oxygen-limited PAH biodegradation ability of these two species can also be assumed. The occurrence of *Acidovorax* in petroleum hydrocarbon–contaminated environments, with elevated concentration of PAHs, is already known just like the PAH bioremediation potential of the genus (Singleton et al. 2018). On the other hand, the implication of *Castellaniella* spp. in PAH biodegradation is not known; only one article discusses weak anthracene degradation ability of the genus (Ntougias et al. 2015).

Based on literature data, it can be assumed that naphthalene was a good choice for the selection and isolation of bacteria capable of degrading other PAHs too. Studies of Wald et al. (2015) and Nwinyi et al. (2013) revealed that the simplest PAH (naphthalene) induced the selection, and allowed the isolation of bacteria capable of degrading besides naphthalene acenaphthene, fluorene, phenanthrene, anthracene, fluoranthene, pyrene, and chrysene. Moreover, based on Table 2, it can be highly assumed that naphthalene-degrading species obtained during this study, particularly *P. veronii*, *A. delafieldii*, and *P. marginalis*, are most probably able to degrade PAHs other than naphthalene. For instance, *Pseudomonas veronii* strain 20a2, enriched on naphthalene from a PAH-contaminated lake sediment sample, was capable of degrading PAHs with three or four rings (high-molecular-weight PAHs, Table 2). In addition, *A. delafieldii* Na3, enriched from a PAH-contaminated soil by using phenanthrene as sole source of carbon and energy, was capable of degrading naphthalene, phenanthrene, chrysene, benz[a]anthracene, and benzo[a]pyrene. Based on the aforementioned, it can be highly assumed that naphthalene-degrading isolates can be representative of bacterial species capable of degrading other PAHs too (Table 2). Nevertheless, the availability of solely naphthalene-degrading organisms is already of a high significance since depending on the quality in the gasoline on average naphthalene contributes 97 ± 1% of the total concentration of the PAHs.

In this study, at least four bacterial strains were isolated with extremely prolific biofilm production ability, isolates HR3.3_3 and HR3.3_4 identified as *P. marginalis*, and isolates HR1.1_1 and HBGN3.3_1 affiliated with *P. veronii*. These organisms were also able to rapidly degrade naphthalene (within only 20 h) and possessed the NDO (2Fe-2S reductase) gene. *P. marginalis* isolates are usually identified as postharvest plant pathogens causing soft rot in fruits and vegetables (Brown 1918; Kudela et al. 2010; El Hassan et al. 2014). Biofilm formation, and petroleum hydrocarbon, as well as PAH biodegradation ability of *P. marginalis* and *P. extremaustralis*/*P. veronii* lineage–related isolates have been reported before (Kim et al. 2018; Daniel et al. 2016; Ghazy et al. 2012; Tribelli and López 2011; Lagacé et al. 2006; Burd and Ward 1996).

The ability of biofilm production is essential for microorganisms. The EPS allows bacteria to attach to particles (soil/sand or other biological or non-biological surfaces) and at the same time protects them against harsh environmental conditions. From a bioremediation perspective, these two traits are extremely important. While the first prevents washout from the contaminated zone of the introduced bacteria with the desired metabolic ability, the second protects the inoculated organisms from both the adverse effects of the endogenous bacterial population and the new environment. In the case of biofilm-based biobarriers, biofilm-producing ability of the used microorganisms is of particular importance (Carreghini et al. 2013). Moreover, according to recent studies, the biofilm production ability may accelerate petroleum hydrocarbon degradation potential of microbes as well. Omarova et al. (2019) proved that microbial biofilms aid in the stabilization of dispersed oil droplets through the formation of biofilm at the oil-water interface. Stabilization of dispersed oil droplets may indirectly accelerate the process of oil biodegradation. According to Dasgupta et al. (2013), biofilm-supported batch cultures of *Pseudomonas* isolates were able to degrade crude oil more effectively than the planktonic cells. As found by Shimada et al. (2012), biofilm-associated *P. stutzeri* T102 cells were more efficient in naphthalene degradation than planktonic cultures.

Eventually, it is worth mentioning that the results obtained in this study may be valid under the applied enrichment conditions. It may happen that under circumstances closer to in situ environmental conditions different results could have been obtained. Similarly to the theory of “great plate count anomaly” (Staley and Konopka 1985) under in vitro conditions, one can hardly mimic the entire in situ conditions. Therefore, during the selective enrichment, the propagation of only those bacteria was promoted which proliferation was favored by the applied enrichment conditions. In this study, the presence of naphthalene and different oxygen levels were only two out of many other selectors originally found in contaminated groundwaters.

Based on the results, it can be concluded that, besides the isolation of BTEX-degraders, the Bugyi-biofilm sample also allowed the isolation of efficient naphthalene-degrading microorganisms. In both aerobic and oxygen-limited naphthalene-amended enrichments, *Pseudomonas* spp. became the most dominant as it has been proven by both cultivation-dependent and cultivation-independent approaches. *Pseudomonas* spp. may be considered bio-indicator species applicable in the detection of the natural attenuation capacity of PAH- or naphthalene-contaminated sites. We supported our previous findings indicating that oxygen availability is one of the main driving factors in the hydrocarbon-contaminated groundwater environments. Two different microbial communities developed during the enrichments. *P. laurentiana* can be an important member of a PAH-degrading community under aerated circumstances while *P. veronii/P. extremaustralis-*, *Acidovorax-*, and *Castellaniella*-related bacteria are most probably capable of PAH biodegradation under oxygen limitation.

A molecular biological tool was developed for the detection of the NDO (naphthalene 1,2-dioxygenase-related 2Fe-2S reductase) genes of Gram-negative bacteria in either genomic or community DNA. The newly designed primer pairs and the developed CODEHOP-PCR technique may be used for monitoring the natural attenuation capacity of PAH-contaminated sites. A bacterial strain collection with prolific biofilm-producing and effective naphthalene-degrading organisms was established. Subsequently, the obtained strain collection can potentially be applied in the development of biofilm-based bioremediation systems for the elimination of PAHs (e.g., biofilm-based biobarriers or SBPs).

## Electronic supplementary material

ESM 1 (PDF 334 kb)
