# Supplementary material for: Aerobic and oxygen-limited naphthalene-amended enrichments induced the dominance of Pseudomonas spp. from a groundwater bacterial biofilm
Source: Appl Microbiol Biotechnol. 2020 May 16;104(13):6023–43. doi: 10.1007/s00253-020-10668-y (PMC7306034; doi:10.1007/s00253-020-10668-y)
Supplement: Supplementary file 1 — (PDF 334 kb) [file 253_2020_10668_MOESM1_ESM.pdf]

Journal Name: Applied Microbiology and Biotechnology

Manuscript Title: Aerobic and oxygen-limited naphthalene amended enrichments induced the dominance of *Pseudomonas* spp. from a groundwater bacterial biofilm

Tibor BENEDEK[a*], Flóra SZENTGYÖRGYI[b], István SZABÓ[b], Milán FARKAS[b], Robert DURAN[c], Balázs KRISZT[b], András TÁNCSICS[a]

[a]*Szent István University, Regional University Centre of Excellence in Environmental Industry, Gödöllő, H-2100, Páter K. u. 1, Hungary;*

[b]*Szent István University, Department of Environmental Protection and Safety, Gödöllő, H-2100, Páter K. u. 1, Hungary;*

[c]*IPREM UMR CNRS 5254, Equipe Environnement et Microbiologie, MELODY Group, Université de Pau et des Pays de l'Adour, Pau, France.*

*Corresponding author: Tel.: +36-(28)-522-000 ext. 1611

Fax: -

E-mail: benedek.tibor@fh.szie.hu

Number of figures: 2

Number of tables: 2

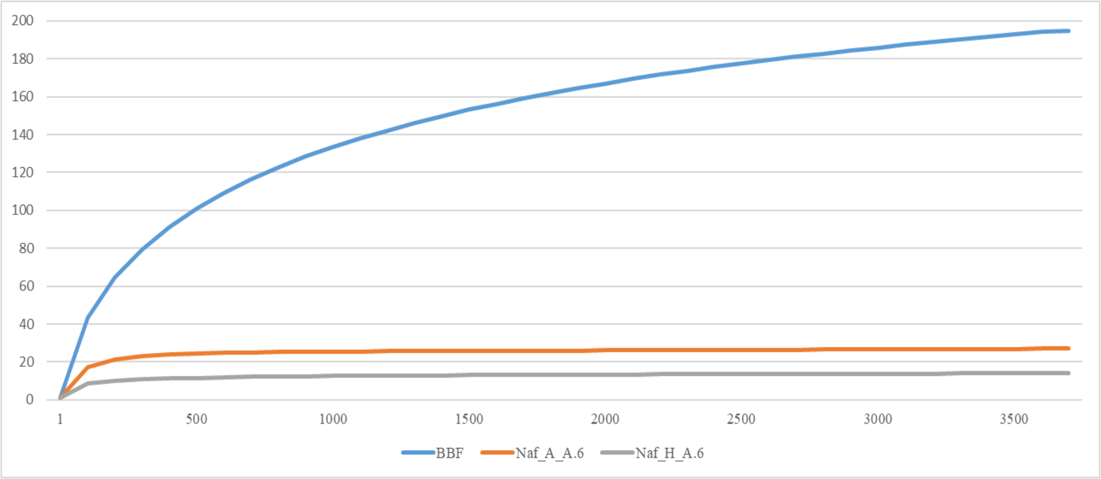

Fig. S1 Rarefaction curves of samples selected for Illumina *16S rRNA* gene based amplicon sequencing

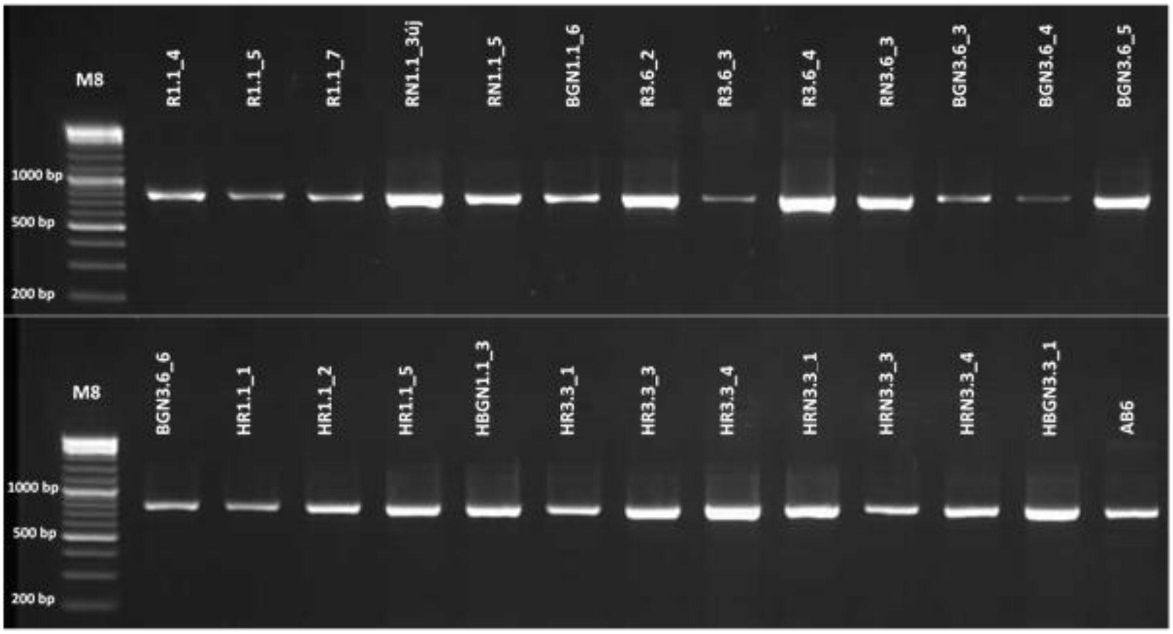

Fig. S2 Detection of NDO related 2Fe-2S reductase component genes of isolates by using the newly designed CODEHOP primer set. 1% agarose gel image. The expected amplicon size is 750 bps.

Table S1 Community composition and bacterial diversity of the investigated samples based on the Illumina *16S rRNA* gene amplicon sequencing dataset

	**Initial Biofilm**	**Aerobic Enrichment (NAF_A_A.6)**	**Oxygen-Limited Enrichment (NAF_H_A.6)**
**Illumina *16S rRNA* amplicon sequencing data**			
Initial sequence read numbers	63828	16683	40765
Sequence read numbers after streaming	44618	3660	40765
Coverage	0.988	0.999	0.999
Shannon diversity_*H*	3.8	1.9	1.3
[a]**Taxonomic composition at the CLASS level** (relative abundance, %)			
Deltaproteobacteria	2.5	6.5	ND
Actinobacteria	0.1	2.8	ND
Holophagae	4.0	ND	ND
Campylobacteria	5.0	ND	ND
Alphaproteobacteria	5.5	4.0	0.1
Bacteroidia	8.0	12.0	ND
Gammaproteobacteria	73.6	73.5	99.9
[a]**Taxonomic composition at the ORDER level** (relative abundance, %)			
Caulobacterales	1.9	0.3	ND
Cytophagales	ND[b]	2.2	ND
Micrococcales	ND	2.8	ND
Sphingobacteriales	ND	3.1	ND
Holophagales	4.0	ND	ND
Xanthomonadales	5.0	7.0	3.1
Enterobacteriales	ND	ND	3.8
Chitinophagales	ND	4.4	ND
Campylobacterales	6.0	ND	ND
Flavobacteriales	6.0	2.6	ND
Myxococcales	ND	6.1	ND
Betaproteobacteriales	76.8	8.9	24.9
Pseudomonadales	0.3	61.4	68.1
[a]**Taxonomic composition at the GENUS level** (relative abundance, %)			
*Xanthomonadaceae*_unclassified	3.0	7.0	0.4
Arcobacter	3.0	ND	ND
Hydrogenophaga	3.2	3.6	ND
Candidimonas	ND	ND	2.0
Achromobacter	ND	ND	2.4
Flavobacterium	2.5	2.6	ND
Simplicispira	3.6	ND	0.4
Burkholderiaceae_unclassified	3.6	ND	ND
Sulfurinomas	3.7	ND	ND
Rhodanobacter	ND	ND	2.7
Geothrix	3.8	ND	ND
Rhodocyclaceae_unclassified	3.0	ND	ND
Lysobacter	3.0	ND	ND
Brevundimonas	3.0	ND	ND
Paenarthrobacter	ND	2.8	ND
Sediminibacterium	0.2	3.4	ND
Aquabacterium	ND	3.6	ND

Enterobacteriaceae_unclassified	ND	ND	3.8
Zoogloea	4.9	ND	ND
Rhodoferax	5.1	ND	ND
Thauera	4.7	ND	ND
Acidovorax	6.7	ND	10.7
Thermomonas	0.2	6.6	0.4
Unknown Labilithrix	ND	7.1	ND
Castellaniella	ND	ND	9.8
Azoarcus	12.5	ND	ND
Sulfuritalea	29.4	ND	ND
Pseudomonas	0.3	61.6	68.1

[a]**Taxonomic composition at the SPECIES level** (relative abundance, %)

Pseudomonas veronii/P. extremaustralis (98.9%)[c]	ND	0.7	63.8
Pseudomonas laurentiana (99.8%)	ND	56.6	ND
Rugosibacter aromaticivorans (96.6%)	17.0	ND	ND
Acidovorax defluvii (99.6%)	4.6	0.4	11.2
Aromatoleum bremense (99.6%)	11.2	ND	ND
Castellaniella caeni (98.7%)	ND	ND	10.4
Thauera humireducens (99.35%)	3.9	ND	ND
Citrobacter portucalensis (99.6%)	ND	ND	3.7
Albidiferax ferrireducens (97.9%)	4.1	ND	ND
Malikia spinosa (99.4%)	2.1	2.9	ND
Unknown *Labilithrix* (92%)	ND	7.1	ND
Luteimonas abyssi v terrae. (95.7%)	ND	5.3	ND
Sulfuritalea hydrogenivorans (98.3%)	3.6	ND	ND
Pseudomonas songnenensis (99.4%)	ND	ND	3.2
Simplicispira psychrophila (98.9%)	2.4	ND	0.6
Geothrix fermentas (96.1%)	2.9	ND	ND
Achromobacter pulmonis v agilis v anxifer (99.6%)	ND	0.7	2.4
Zoogloea ramigera (97.0%)	2.6	ND	ND
Sulfurimonas gotlandica (95.9%)	2.4	ND	ND
Rhodanobacter thiooxydans (99.4%)	ND	ND	2.3
Aquabacterium parvum (98.2%)	ND	3.3	ND
Sediminibacterium goheungense (97.6%)	0.1	3.0	ND
Paenarthrobacter nitroguajacolicus (99.8%)	ND	2.5	ND
Flavobacterium buctense (97.4%)	ND	2.0	ND

[a] – bacteria with relative abundance equal or higher than 2% at least in one of the samples are shown
b – Non-detectable
[c] – *16S rRNA* gene sequence similarities according to the EzTaxon-e database are shown in parentheses (http://eztaxon-e.ezbiocloud.net/)

Table S2 Absorbance values measured at 550 nm for each isolate, obtained during the microplate assay

	Incubation time (hours)					
	24		48		72	
Samples	Mean absorbance measured at 550 nm	STD	Mean absorbance measured at 550 nm	STD	Mean absorbance measured at 550 nm	STD
**Negative c.**	0.13	0.04	0.13	0.04	0.13	0.04
**R1.1_4**	0.14	0.01	0.15	0.01	0.17	0.05
**R1.1_5**	0.16	0.01	0.14	0.00	0.19	0.02
**R1.1_7**	0.62	0.02	0.29	0.01	0.66	0.23
**RN1.1_1**	0.65	0.02	0.36	0.02	0.46	0.01
**RN1.1_3 uj**	0.30	0.01	0.64	0.04	0.56	0.02
**RN1.1_4F**	0.22	0.09	0.16	0.00	0.13	0.00
**RN1.1_5**	0.14	0.00	0.13	0.01	0.15	0.02
**RN1.1_7**	0.75	0.06	0.31	0.03	0.19	0.01
**BGN1.1_2**	0.25	0.00	0.34	0.01	1.43	0.13
**BGN1.1_3**	0.32	0.14	0.17	0.01	0.15	0.02
**BGN1.1_4**	0.15	0.02	0.15	0.01	0.19	0.05
**BGN1.1_5**	0.15	0.01	0.22	0.02	0.23	0.07
**BGN1.1_6**	0.19	0.08	0.15	0.00	0.17	0.01
**BGN1.1_8**	0.15	0.02	0.16	0.02	0.24	0,07
**R3.6_1**	0.22	0.01	0.28	0.16	0.35	0.12
**R3.6_2**	0.31	0.02	0.25	0.02	0.42	0.07
**R3.6_3**	0.26	0.02	0.21	0.02	0.44	0.14
**R3.6_4**	0.43	0.05	0.31	0.13	0.26	0.02
**RN3.6_1**	0.16	0.01	0.18	0.02	0.21	0.01
**RN3.6_3**	0.51	0.03	0.25	0.01	0.23	0.02
**RN3.6_4**	0.32	0.04	0.24	0.01	0.30	0.05
**BGN3.6_1**	0.18	0.01	0.19	0.02	0.22	0.03
**BGN3.6_2**	0.29	0.01	0.38	0.10	1.01	0.03
**BGN3.6_3**	0.19	0.01	0.80	0.03	1.67	0.05
**BGN3.6_4**	0.26	0.02	0.19	0.01	0.17	0.01
**BGN3.6_5**	0.44	0.02	0.25	0.02	0.28	0.00
**BGN3.6_6**	0.22	0.02	0.71	0.04	0.99	0.13
**HR1.1_1**	2.86	0.18	2.68	0.12	2.94	0.24
**HR1.1_2**	0.87	0.06	0.37	0.02	0.50	0.14
**HR1.1_5**	0.73	0.03	0.76	0.09	0.99	0.37
**HBGN1.1_3**	0.26	0.04	0.17	0.01	0.23	0.02
**HBGN1.1_4**	0.59	0.04	0.96	0.09	1.45	0.20
**HR3.3_1**	0.45	0.06	0.91	0.09	1.35	0.09
**HR3.3_3**	2.68	0.23	3.22	0.13	3.57	0.06
**HR3.3_4**	2.64	0.25	3.39	0.42	3.88	0.01
**HR3.3_5**	0.38	0.02	0.21	0.03	0.38	0.06
**HRN3.3_1**	0.94	0.08	0.98	0.11	1.26	0.06

**HRN3.3_3**	1.25	0.17	0.94	0.16	1.28	0.11
**HRN3.3_4**	0.58	0.02	1.46	0.07	2.22	0.07
**HBGN3.3_1**	1.19	0.03	3.39	0.22	3.96	0.01
**A_C**	0.14					
**2 x A_C**	0.28					
**4 x A_C**	0.56					

A_C – cut-off absorbance value for the microplate assay, three standard deviations above the mean absorbance of the negative control.
2 x A_C – level of *weakly adherent*
4 x A_C – level of *strongly adherent*
STD – standard deviation
